# Transcriptome Analysis Reveals Candidate Genes involved in Blister Blight defense in Tea (*Camellia sinensis* (L) Kuntze)

**DOI:** 10.1038/srep30412

**Published:** 2016-07-28

**Authors:** Kuldip Jayaswall, Pallavi Mahajan, Gagandeep Singh, Rajni Parmar, Romit Seth, Aparnashree Raina, Mohit Kumar Swarnkar, Anil Kumar Singh, Ravi Shankar, Ram Kumar Sharma

**Affiliations:** 1 Biotechnology Department, CSIR-Institute of Himalayan Bioresource Technology, Palampur, Himachal Pradesh, India, 176061.

## Abstract

To unravel the molecular mechanism of defense against blister blight (BB) disease caused by an obligate biotrophic fungus, *Exobasidium vexans*, transcriptome of BB interaction with resistance and susceptible tea genotypes was analysed through RNA-seq using Illumina GAIIx at four different stages during ~20-day disease cycle. Approximately 69 million high quality reads were assembled *de novo*, yielding 37,790 unique transcripts with more than 55% being functionally annotated. Differentially expressed, 149 defense related transcripts/genes, namely defense related enzymes, resistance genes, multidrug resistant transporters, transcription factors, retrotransposons, metacaspases and chaperons were observed in RG, suggesting their role in defending against BB. Being present in the major hub, putative master regulators among these candidates were identified from predetermined protein-protein interaction network of *Arabidopsis thaliana*. Further, confirmation of abundant expression of well-known RPM1, RPS2 and RPP13 in quantitative Real Time PCR indicates salicylic acid and jasmonic acid, possibly induce synthesis of antimicrobial compounds, required to overcome the virulence of *E. vexans*. Compendiously, the current study provides a comprehensive gene expression and insights into the molecular mechanism of tea defense against BB to serve as a resource for unravelling the possible regulatory mechanism of immunity against various biotic stresses in tea and other crops.

Tea (*Camellia sinensis* (L) Kuntze) is one of the most popular non-alcoholic beverage crops, globally. It is consumed everyday by millions of people worldwide for its biologically active polyphenols, vitamins, flavanones, catechins and medicinal properties^1^,^2^,^3^. While genomic resources are essential to gain insights of various pathways for dissection of complex traits, it is only very recently that few transcriptomic studies on tea have been performed^4^,^5^,^6^, with no such study in response to biotic stresses. Being a perennial plant, tea comes across a wide range of abiotic and biotic stresses during its life span. Among the biotic stresses, fungal pathogens are the most prevalent, causing severe crop loss annually^7^,^8^. Furthermore, leaf diseases are among the major bottlenecks as commercial tea production is mainly dependent on young succulent leaves^9^.

Blister Blight (BB) disease caused by a basidiomycete obligate biotrophic pathogen *Exobasidium vexans* Massee, is amongst the most serious leaf diseases, significantly affecting commercial production in major tea producing countries, including India, Indonesia, Sri Lanka and Japan. This pathogen mainly attacks young succulent, harvestable tender leaves that not only cause more than 40% total yield loss^10^, but also affects the tea quality significantly by reducing total phenols and catechin content^11^,^12^. Management of the disease faces serious challenges of short but multiple disease cycles with several generations within a single crop season, therefore, requires repeated applications of fungicides^13^,^14^. Although, the application of protectant and eradicant fungicides have shown encouraging results for controlling BB, however, plants face a serious problem of phytotoxicity and fungicide residues. Available bio-controls derived from antagonists namely *Trichoderma harzianum, Gliocladium virens, Serratia marcescens, Pseudomonas fluorescens and Bacillus subtilis* were not found to be very effective^15^,^16^. Furthermore, genetic improvement of resistance against blister blight disease through conventional approaches has suffered due to rare availability of resistant tea accessions, highly heterozygous nature, self-incompatibility and long gestation period of tea^17^,^18^,^19^.

Well understood immune system of *Arabidopsis* and other crop plants suggests that biotrophic pathogens after entering through stomata proliferate in intercellular spaces and obtain nourishment through specialized haustoria. These pathogens, reduce plant immunity by delivering effectors into plant cells. Unlike mobile defender cells and somatic adaptive immune system reported in animals, plants depend on innate/acquired immunity of each cell and systemic signalling from infection sites *via* indirect activation of many resistances (R) genes through well-developed guard hypothesis^20^. In the plant plasma membrane, Pattern Recognition Receptors (PRR) recognize the Microbial/Pathogen Associated Molecular Pattern (MAMP/PAMP) and also activate other Nucleotide Binding (NB) and Leucine Rich Repeats (LRRs). Disease resistance in plant occurs by induction of several resistance proteins and defense enzymes, which act as major immune regulators through physiological and biochemical alterations^21^,^22^,^23^. Additionally, WRKY, NAM transcription factors, LTR retrotransposons, chaperons and metacaspases are among the major defense regulators^20^,^24^, wherein, WRKY and NAM transcription factors play significant role in large scale transcriptional reprogramming by binding to promoter elements of defense related genes and regulating their expression during plant immunity^25^,^26^,^27^. Transcriptome analyses have revealed that putative sites and sequence motifs, ubiquitously conserved in upstream regions of genes are up-regulated during SAR or R-mediated basal defense^28^,^29^. Moreover, activation of several retrotransposons during disease transition elicits defense responses and defense gene activation^30^,^31^. Although, few genes regulating blister blight resistance have been identified^32^, but genome wide transcriptome study to understand the global molecular basis of the immune system against BB has not been elucidated in tea, so far.

In this study, the global gene expression pattern was analysed for the first time using high-throughput Illumina sequencing of young leaf tissues during different stages of BB transition in resistant (RG) and susceptible (SG) genotypes. Comparative transcriptome analysis of RG and SG leads to the identification of putative pathways, genes and their interactions, and suggests good candidates involved in BB defense in tea. Comprehensive efforts of the current study jointly with conventional breeding techniques would thus accelerate genetic improvement of tea and other perennial crops.

## Results

### Transcriptome sequencing

**Figure 1:**
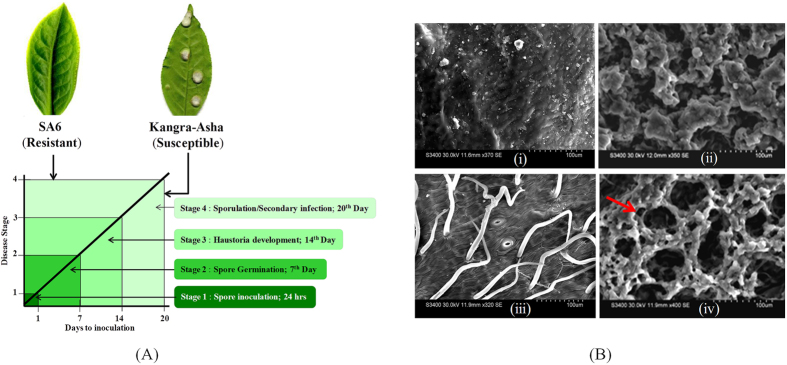
Schematic representation of transcriptome analysis during Blister Blight (BB) transition infected tea leaves. (**A**) Four successive stages of mRNA extraction each from FTL of susceptible (SG; Kangra Asha) and resistant (RG; SA6) tea genotype interaction during 20 days life span of *E. vexan*. Designation of the eight samples was depicted in RG and SG genotypes. (**B**) Scanning electron microscopy of Stage 4 of RG (i & iii) and SG (ii & iv), arrow indicate the hypertrophy/bursting of blister lesion consisting of dense hyphae beneath the lower leaf epidermis in SG at S4.

**Figure 2:**
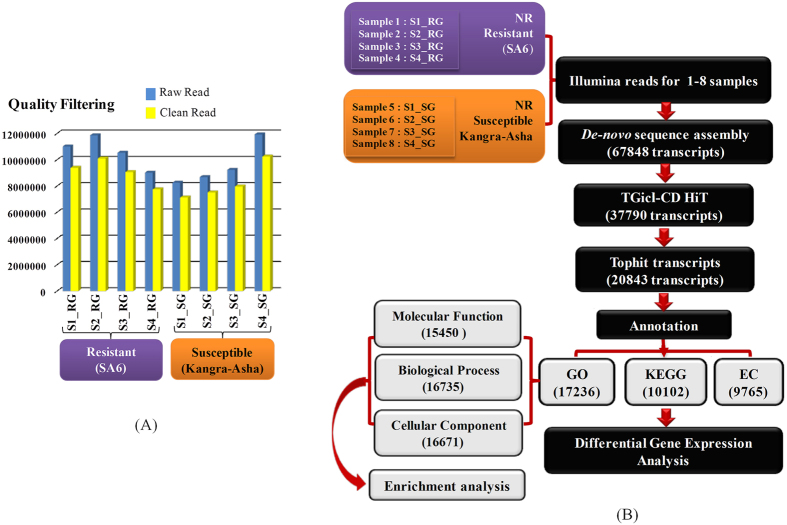
Summary of transcriptome data and analysis approach followed to dissect the defense mechanism against BB in tea. (**A**) Sample wise raw/clean reads obtained in SG and RG. (**B**) Workflow of *de novo* assembly and annotation details of transcriptome data generated in illumina sequencing.

To dissect the molecular mechanism of defense against BB, eight young leaf samples (first two leaves; FTL) of four successive stages from susceptible (SG) and resistant (RG) genotypes was collected during disease progression. Considering the 20-day lifespan of *E. vexans*^8^, four successive stages were categorized as exobasidiospore inoculation or landing of exobasidiospores on the upper surface of leaves [24 hrs post inoculation (PI), Stage1 (S1)]; penetration/germination of spores inside the host tissue [7^th^ day PI; Stage2 (S2)], haustoria and mycelial development [14^th^ day PI; Stage3 (S3)] and sporulation of exobasidiospore [20th day PI; Stage 4 (S4)] (Fig. 1A). Progression of BB disease confirmed through SEM analysis, clearly revealed hypertrophy/bursting of blister lesion consisting of dense hyphae beneath the lower leaf epidermis at SG_S4 (Fig. 1B). To cater sample bias, five subsamples were collected at each stage of RG & SG; independently processed for RNA isolation and pooled at equimolar concentration for making RNA-seq libraries. Transcriptome sequencing of 8 different FTL libraries generated 42,474,318 (RG) and 38,132,457 (SG) paired end (PE) raw reads. Quality filtering and removal of fungal specific reads by mapping to phylogenetically related fungal genome database of *Exobasidium vaccinia* (http://genome.jgi.doe.gov), 36,339,509 and 32,887,574 high quality reads of RG and SG, respectively were achieved (Fig. 2A). To increase the total coverage, frequency and average transcript length, overall 69227083 PE reads obtained from eight libraries were pooled before assembly. Best primary assembly of short reads obtained at a k-mer size of 21 nucleotides^6^, which yielded 67848 primary assembled transcripts with an average length of 1087.58 bp and N50 about 1731. In all the assembly steps, the minimum length cutoff for assembling transcripts was followed as described by Gahlan *et al*.^33^. The raw reads derived from Illumina GAIIx of all the analysed samples have been deposited in National Centre for Biotechnology Information (NCBI) Sequence Read Archive (SRA) with accession number SRP067826 under BioProject, PRJNA306068.

### Homology search and functional annotation

A total of 37790 unique transcripts was generated using a hierarchical clustering approach involving TGICL-CAP3 and CD-HIT^33^ from the primary assembled transcripts, which were further utilized for downstream analysis to dissect a BB defense (Fig. 2B, Table 1). BLASTX^34^ search of 37790 transcripts against NCBI non-redundant (NR) protein sequence database, annotated 20843 transcripts with significant hits. Annotation with Gene Ontology (GO)^35^, Kyoto Encyclopedia of Genes and Genomes (KEGG)^36^,^37^, Enzyme Commission Codes (EC)^36^ and Plant Transcription Factor Database (PlantTFDB)^38^ for each query sequence identified 17237, 10102, 9765, 11515 transcripts, respectively (Fig. 2B; Supplementary Table S1).

### Global gene expression dynamics of defense against BB

**Figure 3:**
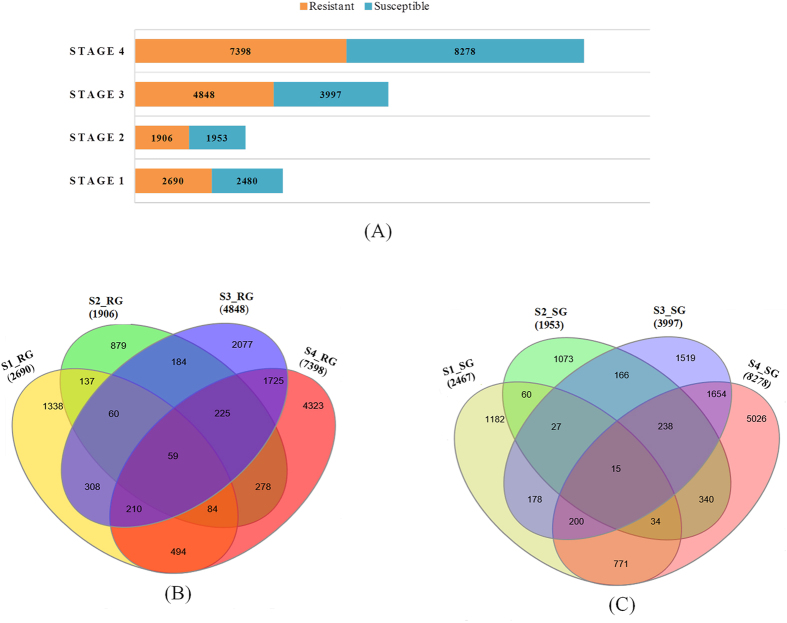
Illustration of **o**verall upregulated transcripts during Blister blight (BB) disease transition. (**A**) Graphical representation of overall upregulated transcripts in both RG and SG; (**B**) number of upregulated transcripts in Resistant genotype (RG) illustrated in the form of Venn Diagram; (**C**) number of upregulated transcripts in Susceptible genotype (SG) illustrated in the form of the Venn Diagram.

Overall transcriptional activity was estimated using edgeR^39^ based on the number of genes up-regulated with fold change ≥2 in RG and SG at different stages during BB progression. Defining the global gene expression dynamics of different sets of genes during disease transition, we found 2690 (S1_RG), 1906 (S2_RG), 4848 (S3_RG) and 7398 (S4_RG) transcripts/genes up-regulated in RG, while, 2467 (S1_SG), 1953 (S2_SG), 3997 (S3_SG) and 8278 (S4_SG) in SG (Fig. 3A). Correlation of RG and SG during the disease transition revealed that greater abundance of transcripts was recorded at S4. In the case of RG during BB transition, 1338, 879, 2077 and 4323 transcripts were uniquely up-regulated in stages S1, S2, S3 and S4 respectively. Interestingly, 59 transcripts were up-regulated commonly in all the stages as illustrated in the venn diagram (Fig. 3B). On the contrary, in SG, 1182, 1073, 1519 and 5026 transcripts were uniquely upregulated in stages S1, S2, S3 and S4 respectively, wherein 15 common transcripts were found to be upregulated in all the stages (Fig. 3C). Despite the higher number of transcripts in SG (8278 transcripts, S4_SG), most of the defense related genes were expressed in S4_RG.

### Gene ontology enrichment and pathway analysis

Gene Ontology (GO)^35^ categories of up-regulated transcripts within each stage of disease progression in RG and SG were identified using annot8r^36^. Biological processes and metabolic pathways GO enrichment analysis revealed that GO terms, namely response to stimulus were found to be highly enriched in S1_RG, where in response to chemical stimulus in the form of organic substance were found to be moderately enriched; response to endogenous stimulus and response to defense of stress category remained enriched. On the other hand, cellular aromatic compound and metabolic process, including salicylic acid metabolism and biosynthetic processes were also found to be enriched in S1_RG (Supplementary Figure S1); while in SG, secondary metabolic process and the flavonoid biosynthesis process were enriched (Supplementary Figure S2). At S2_RG, response to stimuli, especially chemical stimulus of biological process was found to be moderately enriched, whereas oxidation reduction (part of metabolic process), cellular respiration (part of cellular metabolic process) and ion transport (cation transport, and transition metal ion transport) were enriched. Additionally, hydrogen peroxide regulation, a part of regulation of oxygen and reactive oxygen species and metabolic process which is positively regulated by a hydrogen peroxide metabolic process were enriched only in RG (Supplementary Figure S3). At S3_RG, major categories including signalling and response to stimulus were found to be enriched, with response to stimulus, including response to organic substance under the category of response to chemical stimulus being highly enriched. Also, response to fungus (response to biotic stimulus) and cellular response to endogenous stimulus were found to be enriched. Furthermore, phenylpropanoid biosynthetic and metabolic process nested in cellular aromatic compounds and metabolic process category was also enriched in S3_RG (Supplementary Figures4), whereas in S3_SG, only chloroplast RNA processing GO accession was enriched (Supplementary Figure S5). Interestingly, at S4_RG, response to stimulus (highly enriched) remained as a major category with maximally enriched biological GO processes. Defense response and cellular response to chemical stimulus (enriched) under the major subcategories namely response to stress and response to chemical stimulus remained moderately enriched, respectively. Jasmonic acid mediated signalling pathway nested in GO sub-category response to endogenous stimulus, including cellular response to jasmonic acid stimulus and endogenous stimulus were also enriched (Supplementary Figure S6). Nevertheless, during S4_SG histone lysine methylation and DNA replication were enriched in metabolic process; cell cycle, cell wall organization or biogenesis, negatively regulating cell cycle process and cell wall metabolic process were also enriched. Similarly, enrichment of multicellular organismal development, negatively regulating system development and organ development were observed and anatomical structure development under developmental process remained moderately enriched (Supplementary Figure S7).

In molecular function GO enrichment analysis, we found the genes such as Oxidoreductase, Catalytic monoxygenase, Aminopropane-1-carboxylate oxidase, Flavanol synthase, NADH dehydrogenase and Xyloglucosyl transferase and Nuclease and Endonuclease involved in various aspects of defense responses were enriched in RG. Of these, catalytic activity was found to be the most significantly enriched at S4_RG while, Oxidoreductase was significantly enriched irrespective of stages. Flavanol synthase at S1_RG, NADH dehydrogenase and Xyloglucosyl transferase at S2_ SG and Nuclease and Endonuclease were enriched at S3_SG.

Overall GO enrichment analysis revealed salicylic acid biosynthesis and metabolism with a secondary metabolic pathway, possibly activate and trigger the primary immune response in S1_RG, while flavonoid synthesis and other defense related genes trigger immunity in S1_SG. As the disease progresses towards S2 & S3, major secondary metabolite pathways, biotic stress controlling enzymes and free radical scavenging processes provides immunity in RG. In S2_SG, oxidoreductase and cell wall strengthening enzyme play role in defense, but later at S3_SG nuclease activates and probably leads to immune system failure. At S4_RG, enriched expression of jasmonic acid, secondary metabolite, biotic stress controlling enzymes and other strong immune responsible genes provide defense in RG, while, expression of cell division and developmental stage regulating genes clearly indicates cell(s) death, initiating new cell division and development in SG.

### Differential gene expression of defense related genes

**Figure 4:**
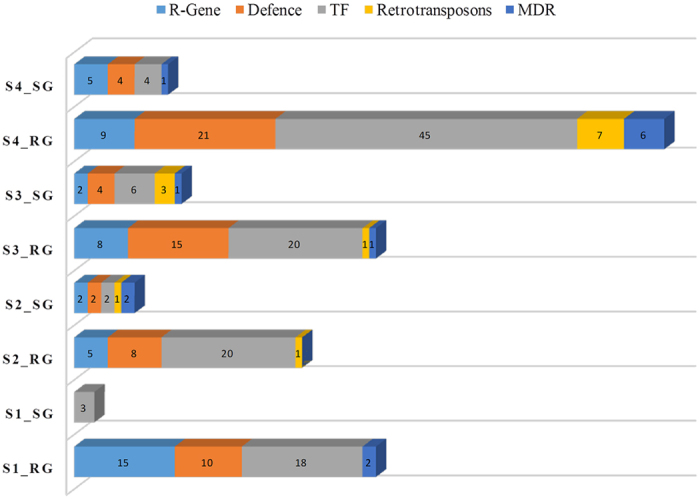
Graphical Representation of immune responsible genes identified in transcriptome data classified into five categories: R-gene, Defense related. TF, Retrotransposons, MDR and Other Defense related genes.

**Figure 5:**
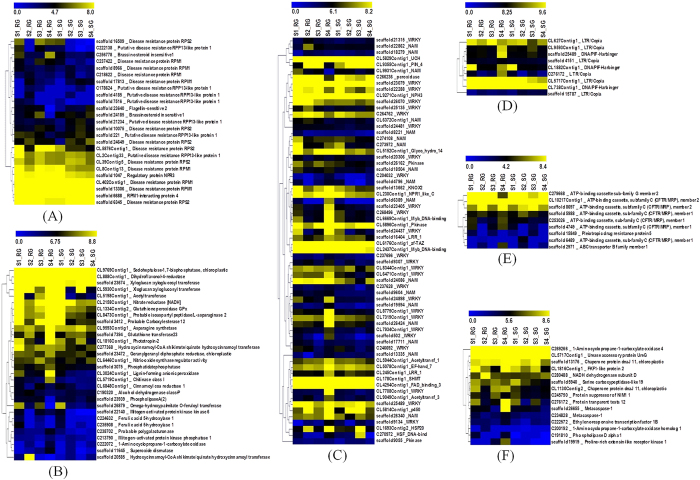
Heat map depicting differential expression profiles of 149 immune related genes at different BB disease transition stages in the RG and SG. (**A**) Resistance genes; (**B**) Defense related enzymes; (**C**) Transcription factors; (**D**) Retrotransposons; (**E**) Multidrug Resistance Defense Transporters. (**F**) Other important defense regulatory genes.

We were specifically interested in identifying the gene expression pattern during BB defense, 149 immune responsible genes classified in KEGG^37^, GO^35^, EC and PlantTFDB^38^ annotations, were studied to elucidate key pathway genes involved in BB defense. These include genes encoding defense related enzymes (30), resistance genes (25), multidrug resistant transporters (9), transcription factors (65), retrotransposons (9), and other defense genes (15) comprising metacaspases (2) and chaperons (2) (Fig. 4). To assess the overall similarity between the immune responsible dataset, the Euclidian distance was calculated between each stage of RG and SG based on transcript abundance values, which further confirms the significance of immune responsive genes in RG at S4 (Fig. 5; Supplementary Table S2).

### Differential expression of resistance (R) genes

The Plant genome encodes many R genes that recognize divergent pathogen effectors and triggers the hypersensitive cell death resistance response. In total, 25 R genes exhibiting significant differential expression during disease transition were identified, among which 14 (S1_RG), 5 (S2_RG) and 9 each at S3_RG and S4_RG were up-regulated. Although, many of these R genes were common irrespective to stage during disease transition, four of the R genes, namely, RIN4, RPM1, RPS2 and RPP13 were most represented (Fig. 4A). A Heat map of R gene clustering indicates that RIN4 possibly regulates other RPM1, RPS2 and RPP13 resistant proteins for early immune development as previously reported in *A. thaliana*^21^. **E**xpression data of these R genes indicate transcriptional dynamics during disease transition.

### Differential expression of defense related enzymes

In total, 1337 enzymes, encoded by tea transcripts during different stages of BB transition were annotated using Enzyme Commission (EC) database. While analysing 30 defense related enzymes, 10 (S1_RG), 8 (S2_RG), 15 (S3_RG) and 21 (S4_RG) were found to be highly up-regulated (Fig. 5B). Of these, 21 up-regulated defense related enzymes at S4_RG with fewer up-regulated irrespective of the BB transition stage, which might account for the long lasting immunity in RG^20^ (Fig. 5B). Up-regulation of acetyl-transferase, xyloglucosyl-transferase, peroxidase and carboxyl-esterase suggests that S4_RG is among the most active stage of BB transition (Supplementary Table S2). Nevertheless, Sedo heptulose 1–7 bisphosphate, a carbon fixing enzyme expressed in both RG and SG possibly provides photosynthate for immune development. Defense enzymes, namely, Xyloglucan:Xyloglucosyl transferase, Dihydroflavonal-4-reductase, Glutathione peroxidase, Glutathione transferase, Asparagine synthase, Quinate hydroxycinnamoyl transferase, Alcohol dehydrogenase, Nitrate reductase, Nitric oxide synthase regulatory protein up-regulated in RG also provide immunity during the early stages of disease transition in SG as revealed by heat map clustering. However, Acetyltransferase, Carboxylesterase, Chitinase, Lignin forming anionic peroxidise, Omega-Hydroxypalmitate-o-feruloyl transferase, ACC oxidase, Polygalacturonase, Superoxide dismutase, Inositol 1,4,5 triphosphate, Mitogen activated protein kinase kinase 6, Cinnamoyl-CoA reductase showed elevated expression in RG.

### Differential expression of defense responsive transcription factor

Transcription factors are key regulatory proteins of biotic and abiotic stress, which mediate the transcriptional regulation^25^. Expression dynamics of transcription factor genes involved in the BB defense identified 66 defense responsive transcription factors exhibiting significant differential expression in RG and SG (Fig. 5C). Among these, WRKY & NAM (NAC domain) families were most represented, while stage wise comparison during BB transition recorded 15, 20, 20 and 44 TFs up regulated in S1_RG, S2_RG, S3_RG and S4_RG, respectively. The highest expression of TFs at S4_RG further confirms the relevance of S4 in BB defense to cope with disease severity due to secondary infection.

### Differential expression of retrotransposons

Retrotransposons remain quiescent during normal condition, but become more active during the onset of various stresses^30^. The new insertion within or flanking to coding regions generates mutation that can lead to changes in the dynamics of gene expression and reshape the genome. It has been reported that LTR retrotransposons are activated during pathogenic infection^40^. In total, 9 transcripts of retrotransposons family exhibited significant differential expression during the BB transition in RG, wherein LTR/Copia were found to be highly upregulated (Fig. 5D). Furthermore, stage wise comparison identified 1 (S2_RG), 2 (S3_RG) and 7 (S4_RG) up-regulated transcripts. Current data indicate that during the higher biotic pressure (S4_RG), more number of up-regulated retrotransposons might play an important role in reshaping the genome leading to immunity in RG.

### Differential expression of multidrug resistance transporters

Plant genome contains a large number of genes encoding putative multidrug resistance transporters, reported to be involved in the transport of a wide range of compounds including auxins, flavonoids, glutathione conjugates, metal chelators, herbicide and antibiotics^41^. We found 9 MDR transporters exhibiting significant differential expression in disease transition (Fig. 5E). Out of these MDR transcripts, 2 in each S1_RG & S3_RG, and 6 in S4_ RG were found to be up-regulated, which further indicates its importance during BB transition, wherein up-regulated MDRs at stage S4 possibly helps in acquiring long lasting immunity of tea in RG.

### Other important defense regulatory genes

Transcripts encoding chaperone, 1-Aminocyclopropane-1-carboxylate oxidase, cytochrome p450, metacaspase and circadian rhythm exhibited significant differential expression during the BB transition with most of them revealing a higher number of up-regulated transcripts (12) in S4_RG (Fig. 5F). Overall differential gene expression of other important defense regulatory genes indicated S4 was the most transcriptionally active stages irrespective of RG and SG.

### Protein-Protein Interactions against BB defense

**Figure 6:**
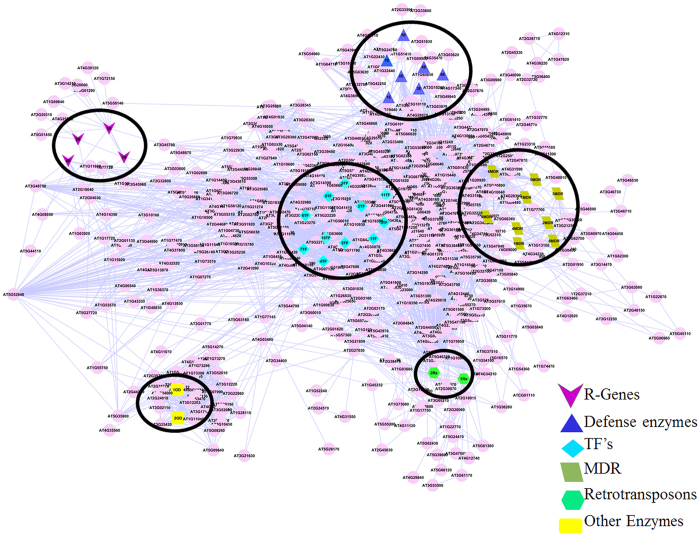
Interactome analysis of putative immune related genes involved in BB defense in tea with a PPI network of *Arabidopsis thaliana*. Categories of Defense related genes (R genes; Defense responsible enzymes; Transcription factors; Retrotransposons; Multidrug Resistance Defense Transporters; Other important defense regulatory genes) depicted with different signs and also indicated in the major hubs PPI network analysis.

For identification of master regulators among the defense related genes, predetermined protein-protein interaction (PPI) network of Arabidopsis was used to predict the key genes/proteins involved in BB defense in tea. Out of 149 defense responsive genes, 126 unique TAIR-IDs were successfully assigned, among which 88 were mapped and found to be interacting with 746 nodes and 3626 edges. Average number of undirected neighbours in the network for each gene was approximately 9.721. On closer analysis, it was observed that 62 of 88 mapped genes were found in the major group containing 661 nodes with average number of neighbours 10.6, while 26 remained in solitary clusters. Based on the interactome, R genes (4), defense enzyme (7), transcription factor (11), reterotransposon (2), multidrug resistance (9) and other defense genes (2) were present in the major hub, hence can be considered as putative key genes for BB defense (Table 2). Furthermore, PPI network analysis revealed that R genes interacting with 35 other genes, defense related enzymes with 125, transcription factors with 149, reterotransposon with 175, multidrug resistant category with 149 genes and other defense related genes with 41 genes; therefore, can be potential candidates for combining of resistance in promising tea genotypes (Fig. 6).

### Experimental validation of differential expression data by qRT-PCR

**Figure 7:**
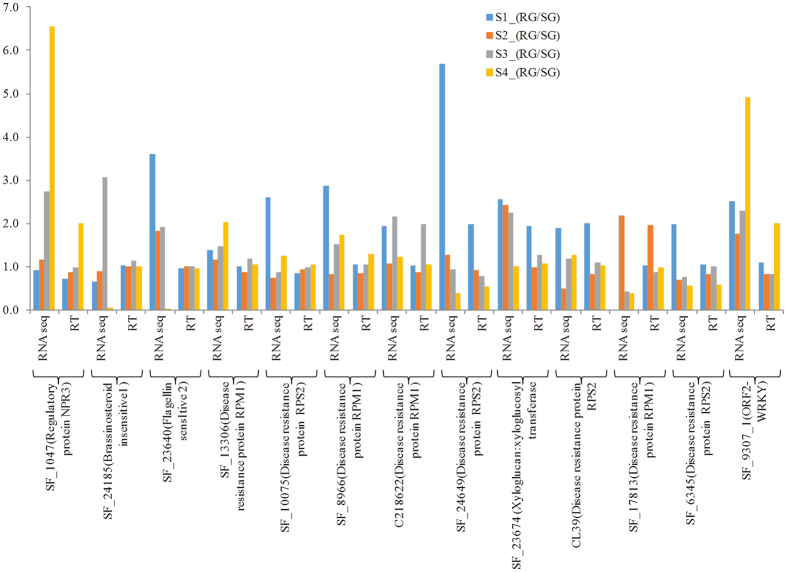
Comparisons between RNA-seq and qRT–PCR expression profiles. Log 2 transformed relative mRNA levels of DEGs by RNA-Seq and analysed by qRT-PCR of defense related genes at different stages during BB progressions in RG and SG.

To validate a differential gene expression pattern obtained through RNA-seq, 13 immune related genes were selected for qRT-PCR analysis (Supplementary Table S3). Among these, expression of transcripts encoding defense related enzymes, multidrug resistant transporters, transcription factors, metacaspases and chaperons obtained in qRT-PCR was found to be largely corresponding with RNA-seq data (Fig. 7). Hence, indicating that RNA-seq approach provided reliable differential gene expression information to understand molecular mechanism of immune response against the BB defense in tea.

## Discussion

It is imperative to use NGS based global transcriptome sequencing approach to identify key pathway genes involved in BB defense in tea as follows in earlier studies for dissecting various biotic stresses^42^,^43^. *E. vexans* Massee is an obligate biotroph thus difficult to study its virulence, and therefore, elucidation of molecular mechanism and identification of key immune gene(s) involved in BB defense in RG could be an appropriate approach to control severe crop loss. Furthermore, identification of diverse R genes will also provide opportunity to study interactions with a wide range of *E. vexans* effectors for ascertaining possible immune signalling during BB defense in tea.

In this study, GO enrichment of the defense related aromatic compound and salicylic acid biosynthesis processes in RG, and secondary metabolite biosynthesis (lignin or phytoalexins like antimicrobial compounds) in S1_SG suggests the activation of the immune system during the initial stages of disease transition irrespective of the RG & SG. The Presence of hydrogen peroxidase activity suggests efficient scavenging of free radicals enabling restricted penetration/germination of spores inside the host tissue at S2_RG. Phenylpropanoid and aromatic compound synthesis perhaps lead to synthesis of antimicrobial compounds that might have provided protection during haustoria and mycelial growth at S3_RG. Concurrently, chloroplast RNA processing, known to reduce photosynthesis, could be responsible for failure of the immune system during S3_SG^44^. Additionally, enrichment of defense response, jasmonic acid stimulus, secondary metabolic process and jasmonic acid mediated signalling during sporulation of exobasidiospore at later stages of BB transition (S4_RG) confirmed that RG developed immunity by jasmonic acid signalling pathway. Although the presence of developmental processes, cell cycle, histone lysine methylation is known to provide small immunity achieved by histone lysine methylation, jasmonic and salicylic acid signalling of immune response, it was totally absent during sporulation of exobasidiospore (S4_SG). Furthermore, flavonol synthesis and secondary metabolite production during S1_SG and presence of xyloglucosyl transferase at S2_SG as evident in the molecular function GO term, might provide a preliminary defense by lignin deposition restricting the entry of pathogen and subsequent spread of disease as reported earlier^45^,^46^, whereas, immune system appeared to have failed in S3_SG due to expression of nucleases and endonucleases activity. In general, molecular function GO analysis revealed a declining trend of immunity during progression of BB from S1_SG to S4_SG. However, up-regulation of monooxygenase and ACC oxidase activity in S4_RG possibly provides defense by removing free radicals and production of ethylene^47^,^48^. Enrichment of defense related genes possibly suggests stronger immunity due to activation of *E. vexans* secondary infection, immune prime cell. Conclusively, the presence of strong immune system machinery might be responsible for long lasting immunity at S4_RG.

Up-regulation of R genes, defense related enzymes, transcription factors, retrotransposons and chaperones during BB transition in RG as revealed in the current study suggests the putative defense mechanism against BB in tea was comparable with model plant *Arabidopsis*. In general, R genes encode five classes of proteins, among which, the NB-LRR (Nucleotide Binding Leucine Rich Repeat) composed of a conserved Nucleotide Binding domain and a variable Leucine Rich Repeat, makes up the largest class^49^. Up-regulation of various R genes during the BB transition in RG possibly increased its recognition capacity to various effectors that limit the pathogen growth by cell wall modification, secondary metabolite production and finally, programmed cell death (hypersensitive response) by activation of salicylic acid signalling pathway, probably involved in tea immunity against BB. In general, disease resistance is governed by specific plant and biotrophic pathogen interaction between pathogen avr gene and resistance R locus alleles^50^. Although, little is known on signalling events required to activate NB-LRR mediated ETI (Effective Triggered Immunity) in tea, presence of few important R genes up-regulated in RG possibly binding to unknown effector(s) of *E.vexans* that might be playing a role in conferring immunity. Among the various R genes reported in *Arabidopsis*, RIN4, a plasma membrane associated protein is guarded by NB-LRR protein and manipulated by AvrRpm1 and AvrB effectors; eventual modification of RIN4 activates RPM1 NB-LRR protein. AvrRpt2 activates a cysteine protease inside the host cell which cleaves RIN4, consequently activating the RPS 2 NB-LRR protein^20^. Up-regulation of RPM1, RPS2-like disease resistance genes in RG suggests guard model might possibly serve as one of the important mechanisms in tea defense against BB. However, separate reverse genetics studies will be required to prove guard hypothesis in tea. Up-regulation of RPP13 (another R gene) in RG insinuates its important role in defense against BB as reported in *A thaliana*^51^. Additionally, higher expression of most of the R genes in RG indicates strongly activated immune machinery, maintained till S4_RG where more biotic pressure exists due to secondary infection of BB.

Penetration of pathogen (hyphae) generally, is prevented due to the presence of a tightly sealed cell wall in RG. This results in depletion of nutrient available to pathogen leading to inhibition of hyphal growth and pathogen toxin diffusion. During the polymerization reaction of cell wall, low molecular weight phenolic compounds which are precursor of lignin and free radicals (ROS) affect the pathogenicity. In response to *E. vexans* infection, lignin metabolism, cellulose synthesis, secondary metabolite synthesis, detoxification of toxic compound, ethylene biosynthesis, free radical scavenger, long lasting immune memory priming responsible enzymes were found to be up regulated at various stages of disease transition and possibly provide immunity as reported previously^20^,^21^. In reference to *A. thaliana,* MPK6 and MPK1, m-RNA and proteins were reported to be accumulated in inactive form in an immune primed cell for long lasting immunity^52^. During secondary cycle/infection of the pathogen, MPK1 and MPK6 were activated in primed cell, thus enabling cell to take strong and rapid action at a very low level of stimulus compared to un-primed cell. Higher MPKK 6 transcript basal level in primed cell irrespective of the disease transition stages might be the reason for strong immune response in RG during secondary infection.

During pathogenic pressure, various transcription factors have been reported to reprogramme transcriptional dynamics of the plant. Among others, WRKY TFs form a large family of regulatory proteins which are known to play an important role in regulating plant immunity^25^. Up-regulated members of WRKY, NAM and other transcription factor families in RG suggests their importance in BB defense^27^. Additionally, R gene activation most likely increases salicylic acid level subsequently leading to NPR1 activation *via* WRKY TF binding upstream of NPR1 gene boxes. The high salicylic acid level might disturb the redox potential of NPR1 protein reducing TGA disulfide bridge, and results a conformational change which allows the TGA family transcription factor to interact with NPR1. TGA-NPR1 complex binds to the promoter of PR gene and possibly provides immunity in RG^25^.

In general, the biotic pressure increases the genome instability^53^. Increased pathogen pressure promotes the formation of R gene by locally changing the epigenetic chromatin landscape to stabilize R gene clusters and allow gene rearrangement. Additionally, transposons activity may contribute to the evolution of R genes and could influence their expression^54^. In our study, up-regulation of LTR and DNA/PIF-Harbinger class of retrotransposons might lead to genome reshuffling and formation of new polymorphic R genes; hence, could be responsible for providing strong immunity. Though the basal level of expression of most retrotransposons was found to be higher in RG irrespective to BB transition, almost all the retrotransposons were activated as a result of elevated *E.vexans* disease pressure due to the secondary infection at S4_RG.

ATP-binding cassette (ABC) transporters mediate the translocation of a wide range of molecules across the biological membrane. ABC transporters are reported to be responsible for the vacuolar import of chlorophyll catabolites and xenobiotics, playing a key role in cellular detoxification by vacuolar sequestration of endogenous or exogenous toxic compounds^55^. Higher basal level of most MDR transporter’s transcripts in RG suggests these perhaps detoxifies the cellular toxin produced by *E. vexans*.

Identification of other important defense related genes such as chaperones and metacaspases might facilitate their conformational changes to induce downstream immune signalling as reported earlier^20^. Chaperone regulates the R protein, eventually leading to programmed cell death (apoptosis) at the site of infection. In plants, tissue or organ apoptosis occurs during normal senescence or response to pathogens. During incompatible interaction, PCD (Programmed Cell Death) occurs and prevents the spreading of pathogen. All these important and defense related regulatory genes were up-regulated at various stages of disease transition with higher basal level of transcripts in RG that may be responsible for regulating tea immunity.

PPI network analysis of 149 defense related proteins with model plant *Arabidopsis* was conducted to reaffirm key proteins modulating BB defense in tea as follows in earlier studies^21^. Being in the PPI network hub and regulating more than 5 other proteins, 35 proteins could thus be designated as key putative proteins involved in BB defense in tea. A Direct association of RIN4 with RPM1 and RPS2 and indirect interaction with NB-ARC domain related to disease resistance indicates that guard hypothesis reported in *Arabidopsis* might be acting in tea defense against BB^20^. Defense related enzymes, namely, superoxide dismutase, mitogen-activated protein kinase kinase-6, nitrate reductase [NADH], phototropin-2, Alcohol dehydrogenase, glutathione peroxidase GPx and cinnamoyl CoA reductase-1, being present in hub possibly regulate other enzymes involved in BB defense. Transcription factors disclose various genes like LRR1, HSP20, WRKY, UCH, HSF_DNA-bind, SHMT, KNOX2, p450, Pkinase and NAM present in network regulating other genes, thus might be playing vital role in BB defense. Among these, NAM (NAC Domain) and WRKY were also found in regulating seconadary metabolites: flavonoid, theanine and caffeine biosynthesis in tea^56^. This suggests that these TFs may regulate the BB defense either directly or indirectly via secondary metabolite biosynthesis. In case of retrotransposons it has been found that 2 genes, LTR/Copia and DNA/PIF-Harbinger associated with 175 other Arabidopsis genes in the network and may have an important role in BB defense. Multi Drug resistant genes, namely ATP-binding cassette, sub-family C (CFTR/MRP), 1 pleiotropic drug resistance protein, 1ABC transporter B family member and 1ABC transporter G family member have shown direct interactions with resistance in predicting network of *Arabidopsis,* hence affirming to be valuable candidates in BB defense in tea. Other genes mapped from identified genes related to defense are metacaspase-1and serine carboxypeptidase-like-19 present in centre regulating various genes in the network. The overall interactome analysis predicted 35 key putative proteins, thus can be categorized as good candidates for regulating BB defense in tea.

## Conclusions

**Figure 8:**
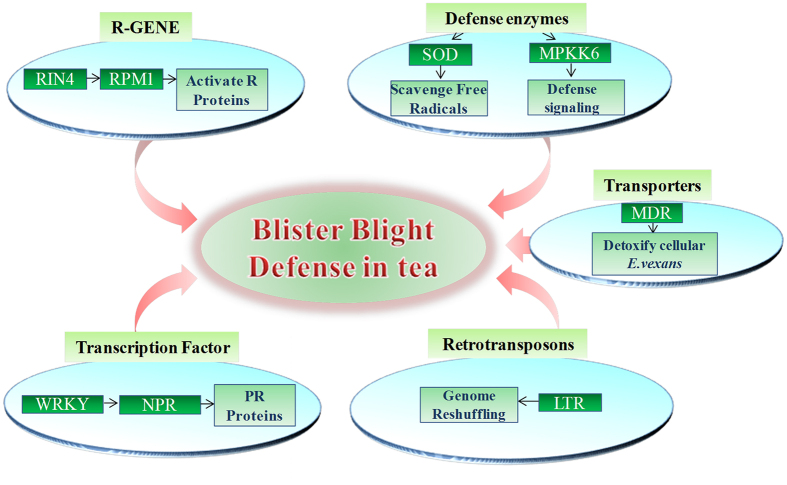
Diagrammatic representation of putative defense mechanism in tea against BB based on different categories of immune related candidates identified in RNA-seq data.

This study, for the first time provides comprehensive defense responsive expressions against the BB defense in tea. An overall analysis initiated after inoculating *E. vexans’* spores revealed activation of various R genes along with defense related enzymes to prevent spread of disease after infection, followed by transporters, involved in overcoming the virulence caused by the pathogen in RG. To cope up with disease severity, retrotransposons are activated and by genome reshuffling possibly produce new type of R gene(s) so that plant is able to recognize a new kind of virulence produced by pathogen. Transcription factors are activated in the plant to further enhance the activity of R and other defense associated genes. Overall, R genes, defense related enzymes, retrotransposons, transcription factors and other defense associated molecules provide immunity in RG (Fig. 8). Furthermore, 149 putative key defense related transcripts/genes identified in expression and PPI network analysis can serve as good candidates for future research and also to be used for combining and characterizing defense system against BB in quality tea clones through molecular breeding and genetic engineering technologies. This information is valuable considering the biotrophic nature of fungi and will provide a powerful approach to identitying potential RG from the random gene pool and to unravel immune regulation of various biotic stresses in tea and other crops.

## Methods

### Plant Material

Tea [*Camellia sinensis* (L.) O. Kuntze] accessions namely SA6 and Kangra-Asha, known source of resistance (RG) and susceptible (SG) response against biotroph fungus *E. vexans* causing blister blight (BB) disease) was used to understand the possible molecular mechanism of defense against BB. SG and RG tea accessions were maintained at the CSIR-Institute of Himalayan Bioresource Technology, Palampur, India (1,300 m altitude; 32°06′ N, 76°33′ E). Vegetatively propagated 3 years old plants of RG and SG were shifted to polyhouse at 22 °C with high humidity (>75%). Plants were sufficiently watered and inoculated with *E. vexans* exobasidiospore suspension at a density of 10^6^ cells/ml at maximum^11^. Leaf tissues (first two leaves; FTL) of RG and SG were collected during the BB transition at different time intervals (24 hour, 7^th^ day, 14^th^ day and 20^th^ day after inoculation; AI), snap-frozen in liquid N_2_ and stored at −80 °C until used for RNA isolation. To avoid the bias in each stage of BB transition, five subsamples were collected for each sample from RG and SG.

### RNA extraction, cDNA library preparation and transcriptome sequencing

Total high quality RNA was extracted using IRIS method^57^. About 100–200 mg of tissue was used to extract the total RNA. The quality and quantity of RNA were assessed with Agilent Bioanalyser using Chip RNA 7500 series II (Agilent Technologies, USA).

cDNA libraries were constructed with approximately 4 μg of RNA (pooled in equimolar concentration from five subsamples)for each sample using the Illumina TruSeq RNA Sample prep Kit v2 LS (Illumina Inc., San Diego, CA) according to the manufacturer’s protocol. The libraries were quantified and checked for quality using Bioanalyser chip DNA 1000 series II. Each library was diluted to 8 picomoles in Elution Buffer (Qiagen) and used for Paired End sequencing using the Illumina Genome Analyser GX II platform (San Diego, CA). Fastq format of PE reads were generated utilizing Illumina’s CASAVA package GERALD tool removing 3 bp from the 3′ ends to minimize sequencing errors. Poor quality reads and reads with lower K-mer frequency was filtered using a FilteR tool (http://scbb.ihbt.res.in/SCBB_dept/filter.php). Transcriptome assembly was carried out using SOAP*denovo,* wherein, high quality reads were cleaved into smaller fragments (21 K-mers) and put together into final contigs using de Bruijn graphs^58^. Further, clustering of assembled contigs/scaffolds was also done to cut down redundancy by merging them using TGICL- CAP3 and CD-HIT-EST at 90% similarity cut-offs^59^, and consensus sequnces were subjected to BLASTX against NR database to get best hits. On the basis of blast tophit dissimilar group clustering were performed using a shell script to identify unigenes, the tophits were searched for a common ID^33^ and grouped together.

### Functional annotation and classification

Assembled transcripts were searched against associated GO^35^ (http://www.geneontology.org), KEGG^37^ (http://www.kegg.jp/kegg/download/) and EC entries. The best hit among the multiple hits for the query transcript sequence was selected on the basis of highest bitscore and E-value (1e–5). Annotation tool, annot8r^36^ was used for GO, EC and KEGG based annotations and statistics. Classification and identification of various transcription factors were performed against the Plant transcription factor database (http://plntfdb.bio.uni-potsdam.de)^38^ using BLASTX with an E-value threshold of 1e–5. GO term enrichment analysis was performed using agriGO^60^.

### Read mapping and expression analysis

Reads Per Kilo base per Million of mapped reads (RPKM), a normalized quantitative procedure for gene expression of RNA Seq data^61^ was used to measure the expression level of each assembled transcript. The filtered reads were uniquely mapped back to various assembled transcripts to estimate the transcript abundance using BOWTIE2^62^. Mapped read counts on transcripts were analysed for differential gene expression based on log2 fold count after normalization using EdgeR^39^. Expression data were collected for each of the transcript for all the eight stages of BB transition in resistant (RG) and susceptible (SG) genotypes. The transcripts with fold change (≥2) irrespective of stages during disease transition were selected for enrichment analysis.

### Validation and expression analysis by RT-PCR

Gene expression results were validated by Real time PCR (RT-PCR). RNA was pre-treated with RNase-free DNase I (Invitrogen, USA) to remove contaminating DNA followed by the cDNA synthesis by using 2.0 μg of total RNA with the help of superscript III (Invitrogen, USA) as per manufacturer’s instructions. PCR conditions, including primer details for RT-PCR are mentioned in Supplementary Table S3. Cycling conditions were optimized to obtain amplification within the exponential phase. Each reaction was carried out triplicates, and 18 s rRNA and RUBISCO were used as an internal control for normalization.

### Interactome analysis of key transcripts associated with BB defense

To further confirm putative master regulators of defense against BB, putative defense responsible differentially expressed transcripts in RG and SG that include R-genes, defense related enzymes, transcription factors, retrotransposons and multidrug resistant transporters were utilized for intractome analysis. Due to limited genomic information (whole genome/transcriptome) in the tea plant, predetermined protein-protein interactions (PPI) network of *Arabidopsis thaliana* (www.arabidopsis.org/index.jsp) was used for mapping of recognized transcripts^51^. Functional and regulatory defense related transcripts were searched with TAIR to find putative targets using BLASTX (1e–5) and mapped to PPI network. A correlation edge was considered as a conserved correlation edge when the correlation between the defense related gene pair in tea was supported by a significant correlation edge between its respective orthologs in the *Arabidopsis thaliana* PPI network (AtPIN) using Cytoscape software (version 2.8)^63^. First neighbour of mapped IDs was selected for predicting their interaction in network to create the regulatory network. Additionally, based on the interactome statistics, putative master regulators were considered to be interacting with at least five other proteins from each defense category.

## Additional Information

**How to cite this article**: Jayaswall, K. *et al*. Transcriptome Analysis Reveals Candidate Genes involved in Blister Blight defense in Tea (*Camellia sinensis* (L) Kuntze). *Sci. Rep.* **6**, 30412; doi: 10.1038/srep30412 (2016).

## Supplementary Material

Supplementary Information

Supplementary Table S1

Supplementary Table S2

## Figures and Tables

**Table 1:** Overview of the *De novo* assembly.

Assembly Statistics	Details
K-mer	21
N 75 length	675
N 50 length	1,731
Minimum length (bp)	325
Maximum length (bp)	11,318
Average length (bp)	1087.58
Total Contigs	37790

**Table 2:** Summarization of protein-protein interaction (PPI) network of key genes of BB defense identified in transcriptome analysis in tea with *Arabidopsis* proteome.

Defense category	Codes	Transcript ID	Arabidopsis ID	No. of Nodes	Description
R-Genes	1R	scaffold6688	AT3G25070	5	RPM1-interacting protein 4
	2R	C237422	AT3G07040	5	Disease resistance protein RPM1
	3R	scaffold10075	AT4G26090	12	Disease resistance protein RPS2
	4R	scaffold6345	AT4G19050	13	Disease resistance protein RPS2
Enzymes	1E	scaffold11645	AT1G08830	36	Superoxide dismutase
	2E	scaffold22140	AT5G56580	35	Mitogen-activated protein kinase kinase 6
	3E	CL2158Contig1	AT1G37130	12	Nitrate reductase [NADH]
	4E	CL1816Contig1	AT1G68050	18	Phototropin-2
	5E	CL6848Contig1	AT1G68540	8	Cinnamoyl CoA reductase 1
	6E	CL1334Contig2	AT2G31570	5	Glutathione peroxidase GPx
	7E	C190320	AT1G77120	11	Alcohol dehydrogenase class-P
Transcription Factors	1TF	scaffold16404_2_ORF1	AT4G33430	21	LRR_1
	2TF	CL1693Contig2_4_ORF2	AT5G12020	16	HSP20
	3TF	scaffold9307_1_ORF2	AT1G80840	5	WRKY
	4TF	CL8344Contig1_6_ORF4	AT4G23810	11	WRKY
	5TF	CL5829Contig1_6_ORF1	AT3G20630	23	UCH
	6TF	C270972_2_ORF1	AT3G22830	13	HSF_DNA-bind
	7TF	CL178Contig1_3_ORF1	AT4G37930	23	SHMT
	8TF	scaffold13662	AT1G62990	15	KNOX2
	9TF	CL5814Contig1	AT1G74110	5	p450
	10TF	scaffold26162	AT2G48010	9	Pkinase
	11TF	scaffold24086	AT5G13180	8	NAM
Retrotransposons	1Re	CL738Contig1	AT1G62750	19	DNA/PIF-Harbinger
	2Re	C276172	AT3G26590	156	LTR/Copia
Multi Drug resistant	1MDR	C253026	AT1G04120	12	ATP-binding cassette, sub-family C (CFTR/MRP), member 1
	2MDR	CL10217Contig1	AT3G59140	14	ATP-binding cassette, subfamily C (CFTR/MRP), member 2
	3MDR	scaffold8697	AT2G47800	12	ATP-binding cassette, subfamily C (CFTR/MRP), member 2
	4MDR	scaffold4749	AT3G13090	15	ATP-binding cassette, subfamily C (CFTR/MRP), member 1
	5MDR	scaffold2971	AT3G28390	20	ABC transporter B family member 1
	6MDR	scaffold6489	AT3G13080	51	ATP-binding cassette, sub-family C (CFTR/MRP), member 1
	7MDR	scaffold15849	AT1G15520	6	Pleiotropic drug resistance protein 5
	8MDR	scaffold5988	AT3G60160	14	ATP-binding cassette, sub-family C (CFTR/MRP), member 1
	9MDR	C279668	AT2G13610	5	ATP-binding cassette sub-family G member 2
Other defence related genes	1OD	C234828	AT1G02170	6	Metacaspase-1
	2OD	scaffold5940	AT4G12910	35	Serine carboxypeptidase-like 19
