# Supplementary material for: Transcriptome Analysis Reveals Candidate Genes involved in Blister Blight defense in Tea (Camellia sinensis (L) Kuntze)
Source: Sci Rep. 2016 Jul 28;6:30412. doi: 10.1038/srep30412 (PMC4964330; doi:10.1038/srep30412)
Supplement: Supplementary Information [file srep30412-s1.pdf]

Supplementary information -SREP-15-38608B

Original Research Article

Transcriptome Analysis Reveals Candidate Genes involved in Blister Blight defense in Tea (*Camellia sinensis* (L) Kuntze)

Kuldip Jayaswall[1,2], Pallavi Mahajan[1], Gagandeep Singh[1], Rajni Parmar[1], Romit Seth[1], Aparnashree Raina[1], Mohit Kumar Swarnkar[1], Anil Kumar Singh[1,3], Ravi Shankar[1], Ram Kumar Sharma[1]*

[1]Biotechnology Department, CSIR-Institute of Himalayan Bioresource Technology, Palampur, Himachal Pradesh, India, 176061

[2]Present Address: ICAR-Directorate of Onion and Garlic Research, Rajguru Nagar, Pune, Maharashtra, India, 410505

[3]Present Address: ICAR-Indian Institute of Agricultural Biotechnology, PDU Campus, IINRG, Namkum, Ranchi-834010, (JH), India

***Correspondence:**

Dr Ram Kumar Sharma
Biotechnology Division
CSIR-Institute of Himalayan Bioresource Technology
Palampur (H.P)
India.
Email: rksharma.ihbt@gmail.com, ramsharma@ihbt.res.in

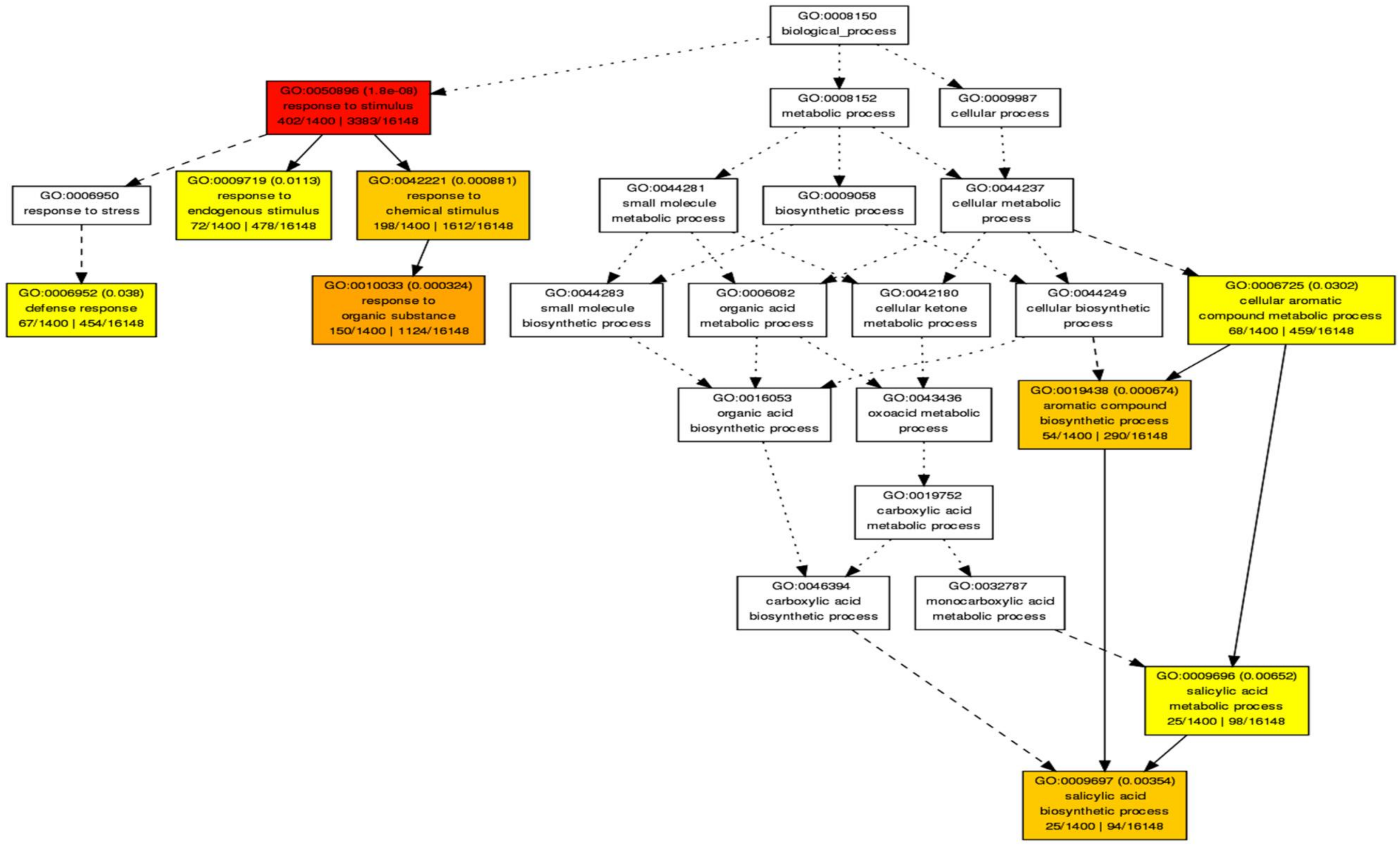
GO:0008150
biological_process
GO:0050896 (1.8e-08)
response to stimulus
402/1400 | 3383/16148
GO:0008152
metabolic process
GO:0009987
cellular process
GO:0006950
response to stress
GO:0009719 (0.0113)
response to
endogenous stimulus
72/1400 | 478/16148
GO:0042221 (0.000881)
response to
chemical stimulus
198/1400 | 1612/16148
GO:0044281
small molecule
metabolic process
GO:0009058
biosynthetic process
GO:0044237
cellular metabolic
process
GO:0006952 (0.038)
defense response
67/1400 | 454/16148
GO:0010033 (0.000324)
response to
organic substance
150/1400 | 1124/16148
GO:0044283
small molecule
biosynthetic process
GO:0006082
organic acid
metabolic process
GO:0042180
cellular ketone
metabolic process
GO:0044249
cellular biosynthetic
process
GO:0006725 (0.0302)
cellular aromatic
compound metabolic process
68/1400 | 459/16148
GO:0016053
organic acid
biosynthetic process
GO:0043436
oxoacid metabolic
process
GO:0019438 (0.000674)
aromatic compound
biosynthetic process
54/1400 | 290/16148
GO:0019752
carboxylic acid
metabolic process
GO:0046394
carboxylic acid
biosynthetic process
GO:0032787
monocarboxylic acid
metabolic process
GO:0009696 (0.00652)
salicylic acid
metabolic process
25/1400 | 98/16148
GO:0009697 (0.00354)
salicylic acid
biosynthetic process
25/1400 | 94/16148

Figure S1

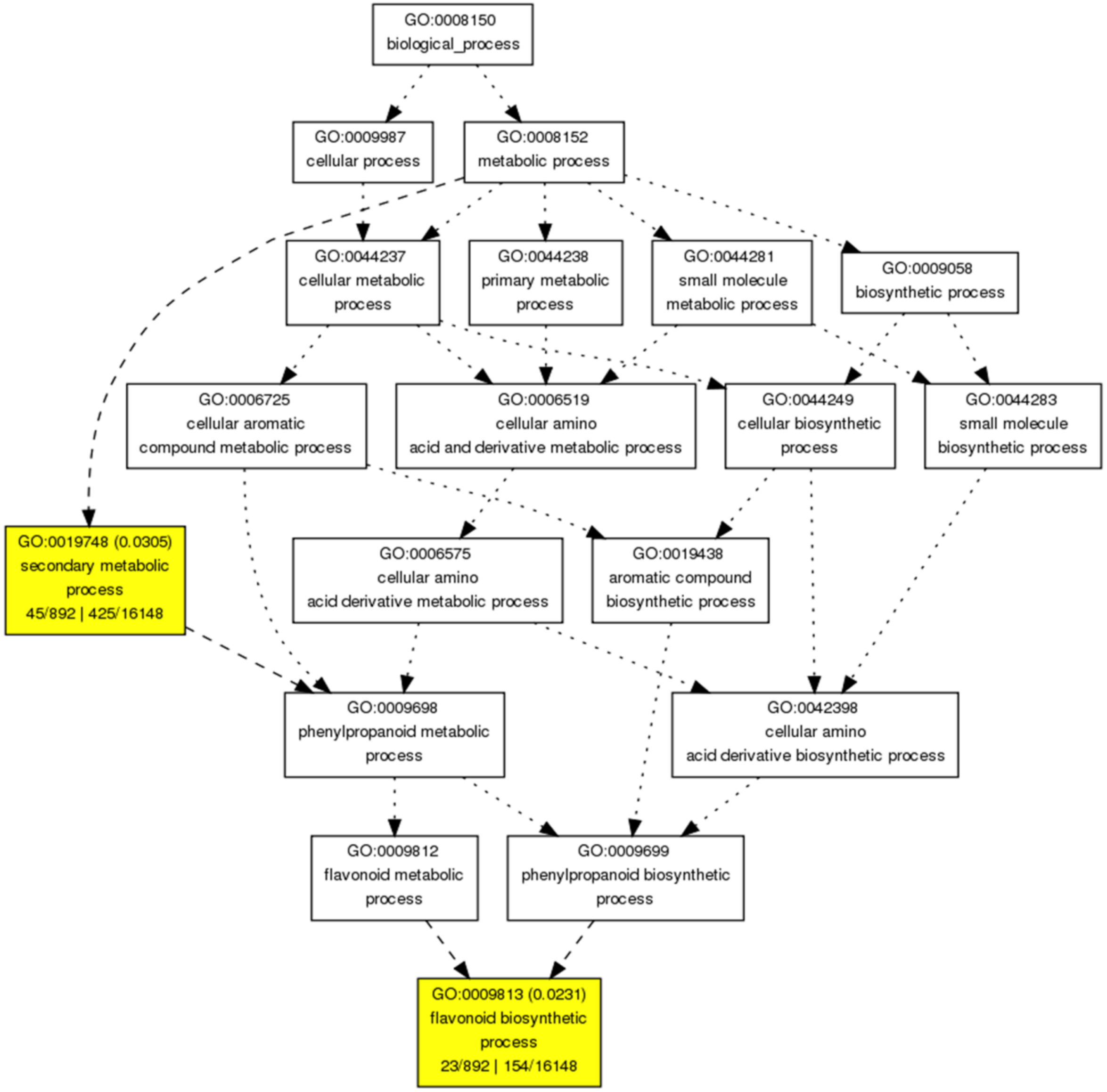

GO:0008150
biological_process
GO:0009987
cellular process
GO:0008152
metabolic process
GO:0044237
cellular metabolic
process
GO:0044238
primary metabolic
process
GO:0044281
small molecule
metabolic process
GO:0009058
biosynthetic process
GO:0006725
cellular aromatic
compound metabolic process
GO:0006519
cellular amino
acid and derivative metabolic process
GO:0044249
cellular biosynthetic
process
GO:0044283
small molecule
biosynthetic process
GO:0019748 (0.0305)
secondary metabolic
process
45/892 | 425/16148
GO:0006575
cellular amino
acid derivative metabolic process
GO:0019438
aromatic compound
biosynthetic process
GO:0009698
phenylpropanoid metabolic
process
GO:0042398
cellular amino
acid derivative biosynthetic process
GO:0009812
flavonoid metabolic
process
GO:0009699
phenylpropanoid biosynthetic
process
GO:0009813 (0.0231)
flavonoid biosynthetic
process
23/892 | 154/16148

Figure S2

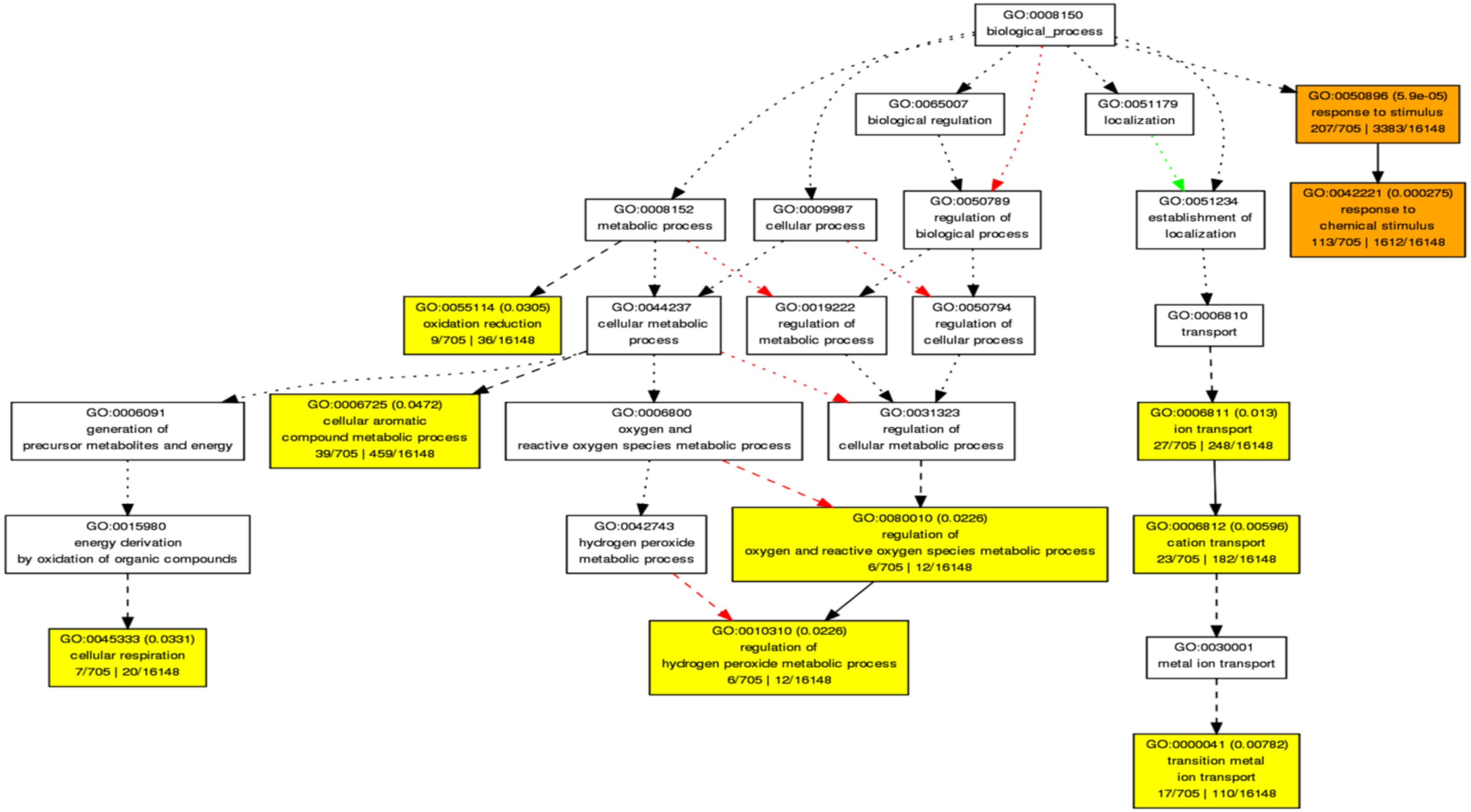
GO:0008150
biological_process
GO:0065007
biological regulation
GO:0051179
localization
GO:0050896 (5.9e-05)
response to stimulus
207/705 | 3383/16148
GO:0042221 (0.000275)
response to
chemical stimulus
113/705 | 1612/16148
GO:0008152
metabolic process
GO:0009987
cellular process
GO:0050789
regulation of
biological process
GO:0051234
establishment of
localization
GO:0055114 (0.0305)
oxidation reduction
9/705 | 36/16148
GO:0044237
cellular metabolic
process
GO:0019222
regulation of
metabolic process
GO:0050794
regulation of
cellular process
GO:0006810
transport
GO:0006091
generation of
precursor metabolites and energy
GO:0006725 (0.0472)
cellular aromatic
compound metabolic process
39/705 | 459/16148
GO:0006800
oxygen and
reactive oxygen species metabolic process
GO:0031323
regulation of
cellular metabolic process
GO:0006811 (0.013)
ion transport
27/705 | 248/16148
GO:0015980
energy derivation
by oxidation of organic compounds
GO:0042743
hydrogen peroxide
metabolic process
GO:0080010 (0.0226)
regulation of
oxygen and reactive oxygen species metabolic process
6/705 | 12/16148
GO:0006812 (0.00596)
cation transport
23/705 | 182/16148
GO:0045333 (0.0331)
cellular respiration
7/705 | 20/16148
GO:0010310 (0.0226)
regulation of
hydrogen peroxide metabolic process
6/705 | 12/16148
GO:0030001
metal ion transport
GO:0000041 (0.00782)
transition metal
ion transport
17/705 | 110/16148

Figure S3

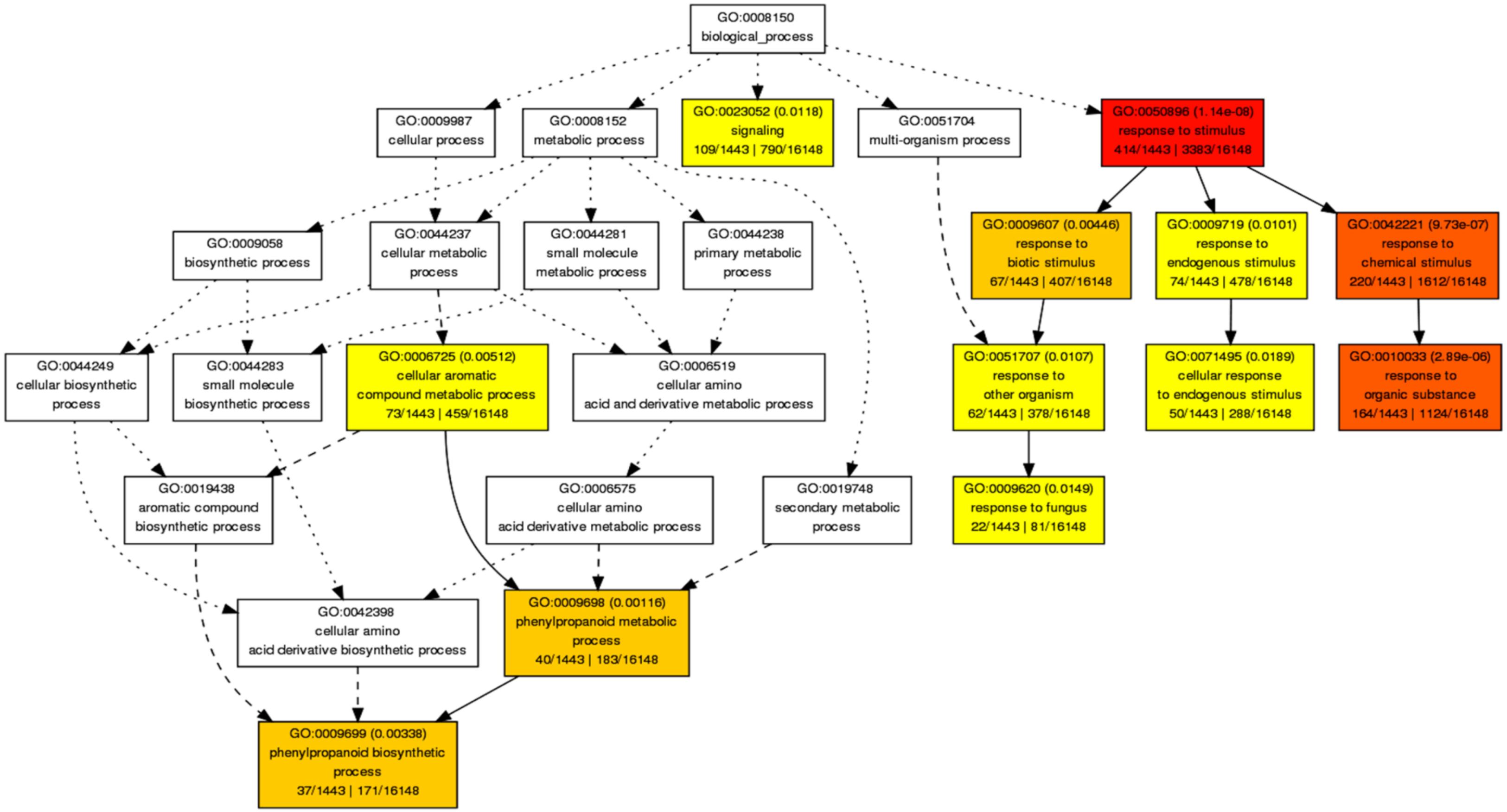
GO:0008150
biological_process
GO:0009987
cellular process
GO:0008152
metabolic process
GO:0023052 (0.0118)
signaling
109/1443 | 790/16148
GO:0051704
multi-organism process
GO:0050896 (1.14e-08)
response to stimulus
414/1443 | 3383/16148
GO:0009058
biosynthetic process
GO:0044237
cellular metabolic
process
GO:0044281
small molecule
metabolic process
GO:0044238
primary metabolic
process
GO:0009607 (0.00446)
response to
biotic stimulus
67/1443 | 407/16148
GO:0009719 (0.0101)
response to
endogenous stimulus
74/1443 | 478/16148
GO:0042221 (9.73e-07)
response to
chemical stimulus
220/1443 | 1612/16148
GO:0044249
cellular biosynthetic
process
GO:0044283
small molecule
biosynthetic process
GO:0006725 (0.00512)
cellular aromatic
compound metabolic process
73/1443 | 459/16148
GO:0006519
cellular amino
acid and derivative metabolic process
GO:0051707 (0.0107)
response to
other organism
62/1443 | 378/16148
GO:0071495 (0.0189)
cellular response
to endogenous stimulus
50/1443 | 288/16148
GO:0010033 (2.89e-06)
response to
organic substance
164/1443 | 1124/16148
GO:0019438
aromatic compound
biosynthetic process
GO:0006575
cellular amino
acid derivative metabolic process
GO:0019748
secondary metabolic
process
GO:0009620 (0.0149)
response to fungus
22/1443 | 81/16148
GO:0042398
cellular amino
acid derivative biosynthetic process
GO:0009698 (0.00116)
phenylpropanoid metabolic
process
40/1443 | 183/16148
GO:0009699 (0.00338)
phenylpropanoid biosynthetic
process
37/1443 | 171/16148

Figure S4

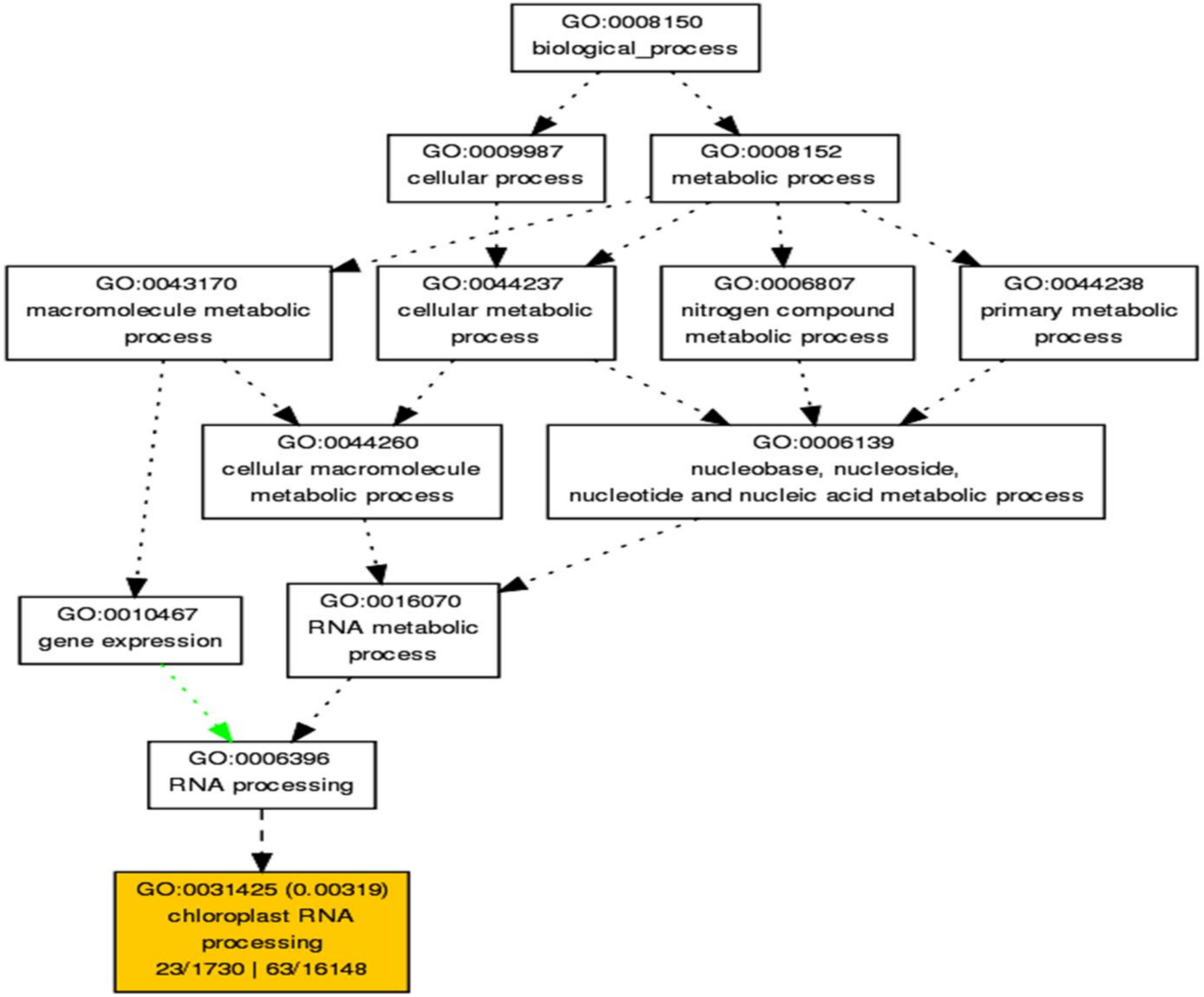
GO:0008150
biological_process
GO:0009987
cellular process
GO:0008152
metabolic process
GO:0043170
macromolecule metabolic process
GO:0044237
cellular metabolic process
GO:0006807
nitrogen compound metabolic process
GO:0044238
primary metabolic process
GO:0044260
cellular macromolecule metabolic process
GO:0006139
nucleobase, nucleoside, nucleotide and nucleic acid metabolic process
GO:0010467
gene expression
GO:0016070
RNA metabolic process
GO:0006396
RNA processing
GO:0031425 (0.00319)
chloroplast RNA processing
23/1730 | 63/16148

Figure S5

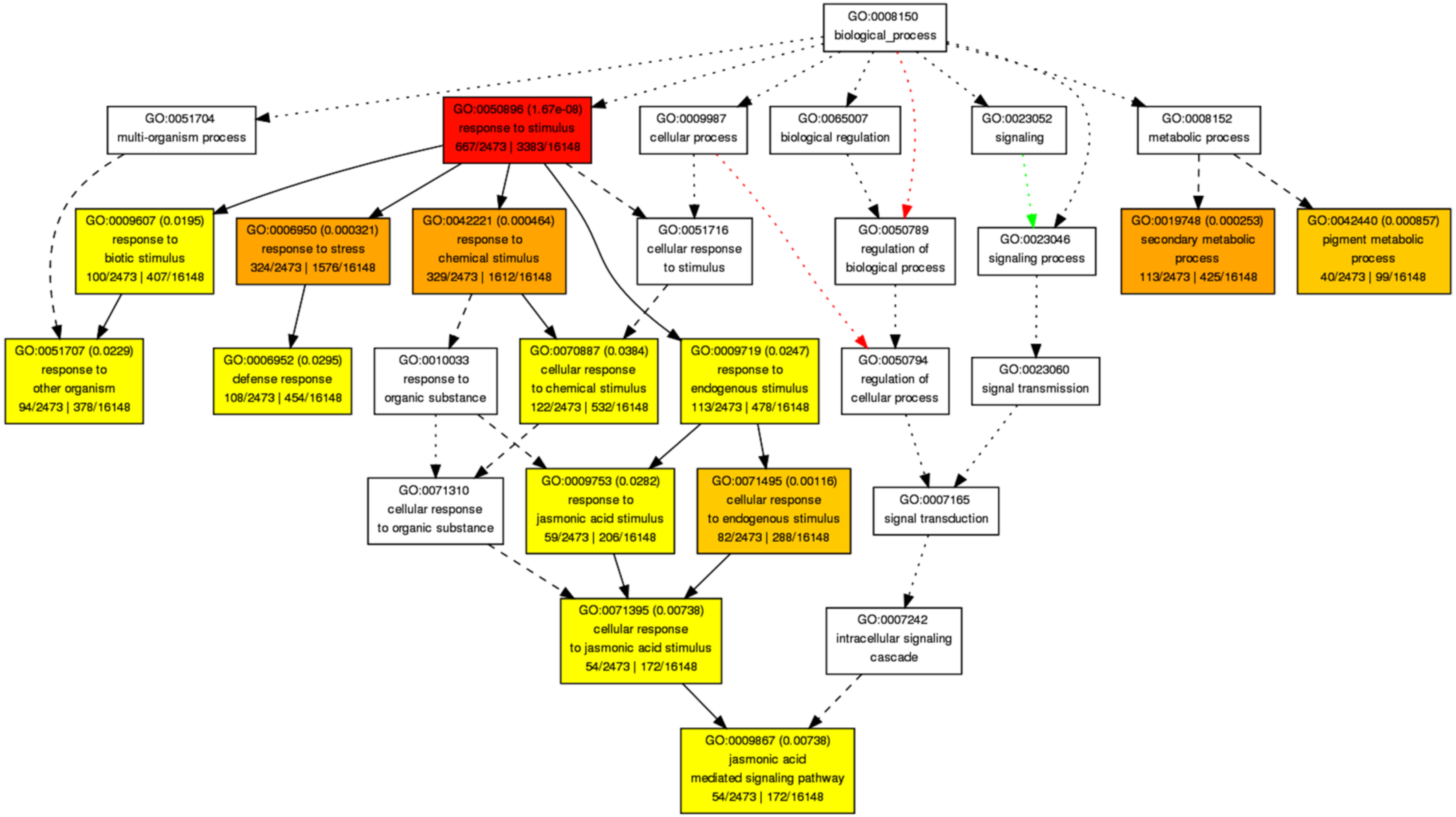

GO:0008150
biological_process
GO:0051704
multi-organism process
GO:0050896 (1.67e-08)
response to stimulus
667/2473 | 3383/16148
GO:0009987
cellular process
GO:0065007
biological regulation
GO:0023052
signaling
GO:0008152
metabolic process
GO:0009607 (0.0195)
response to
biotic stimulus
100/2473 | 407/16148
GO:0006950 (0.000321)
response to stress
324/2473 | 1576/16148
GO:0042221 (0.000464)
response to
chemical stimulus
329/2473 | 1612/16148
GO:0051716
cellular response
to stimulus
GO:0050789
regulation of
biological process
GO:0023046
signaling process
GO:0019748 (0.000253)
secondary metabolic
process
113/2473 | 425/16148
GO:0042440 (0.000857)
pigment metabolic
process
40/2473 | 99/16148
GO:0051707 (0.0229)
response to
other organism
94/2473 | 378/16148
GO:0006952 (0.0295)
defense response
108/2473 | 454/16148
GO:0010033
response to
organic substance
GO:0070887 (0.0384)
cellular response
to chemical stimulus
122/2473 | 532/16148
GO:0009719 (0.0247)
response to
endogenous stimulus
113/2473 | 478/16148
GO:0050794
regulation of
cellular process
GO:0023060
signal transmission
GO:0071310
cellular response
to organic substance
GO:0009753 (0.0282)
response to
jasmonic acid stimulus
59/2473 | 206/16148
GO:0071495 (0.00116)
cellular response
to endogenous stimulus
82/2473 | 288/16148
GO:0007165
signal transduction
GO:0071395 (0.00738)
cellular response
to jasmonic acid stimulus
54/2473 | 172/16148
GO:0007242
intracellular signaling
cascade
GO:0009867 (0.00738)
jasmonic acid
mediated signaling pathway
54/2473 | 172/16148

Figure S6

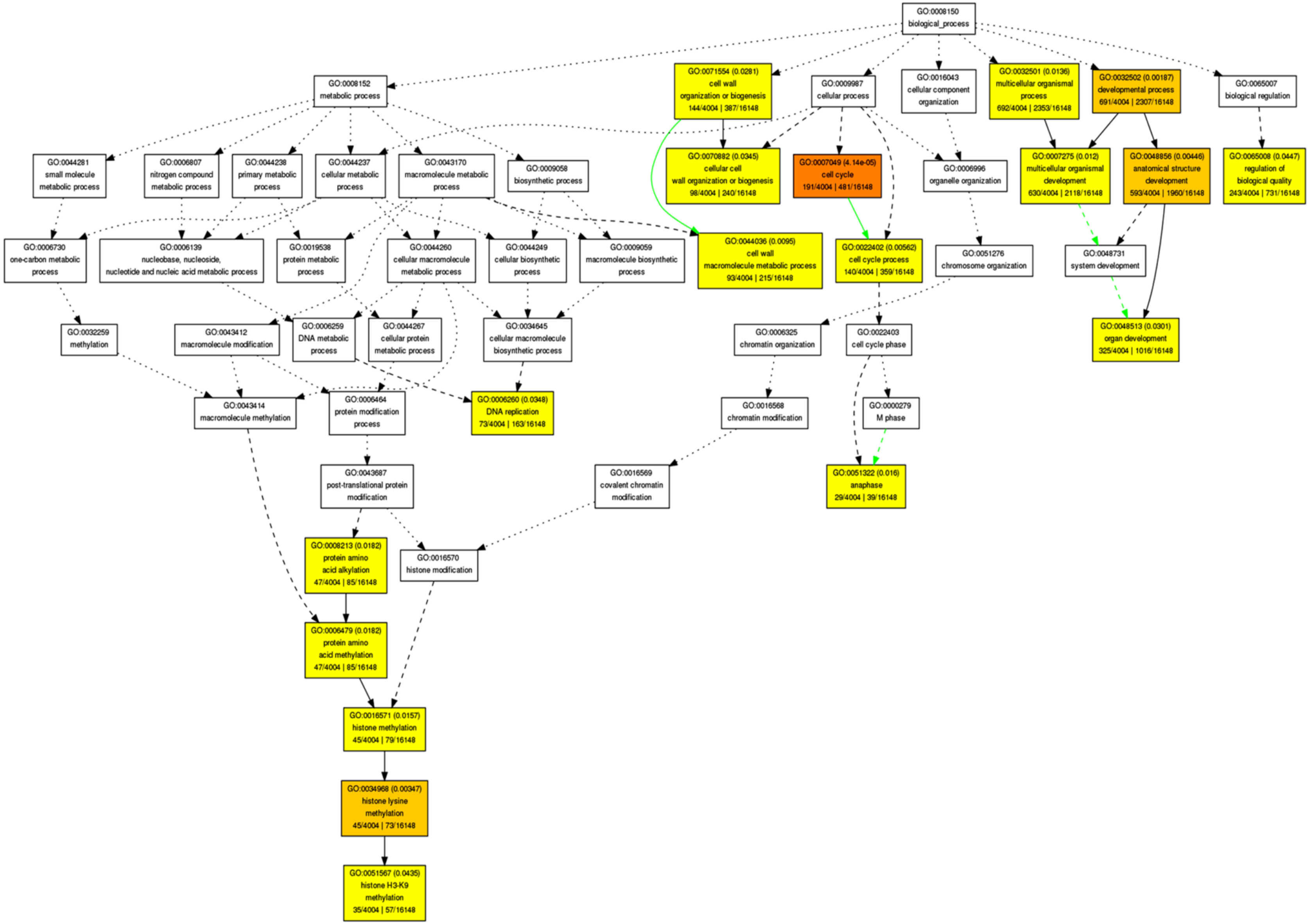
GO:0008150
biological_process
GO:0008152
metabolic process
GO:0071554 (0.0281)
cell wall
organization or biogenesis
144/4004 | 387/16148
GO:0009987
cellular process
GO:0016043
cellular component
organization
GO:0032501 (0.0136)
multicellular organismal
process
692/4004 | 2353/16148
GO:0032502 (0.00187)
developmental process
691/4004 | 2307/16148
GO:0065007
biological regulation
GO:0044281
small molecule
metabolic process
GO:0006807
nitrogen compound
metabolic process
GO:0044238
primary metabolic
process
GO:0044237
cellular metabolic
process
GO:0043170
macromolecule metabolic
process
GO:0009058
biosynthetic process
GO:0070882 (0.0345)
cellular cell
wall organization or biogenesis
98/4004 | 240/16148
GO:0007049 (4.14e-05)
cell cycle
191/4004 | 481/16148
GO:0006996
organelle organization
GO:0007275 (0.012)
multicellular organismal
development
630/4004 | 2118/16148
GO:0048856 (0.00446)
anatomical structure
development
593/4004 | 1960/16148
GO:0065008 (0.0447)
regulation of
biological quality
243/4004 | 731/16148
GO:0006730
one-carbon metabolic
process
GO:0006139
nucleobase, nucleoside,
nucleotide and nucleic acid metabolic process
GO:0019538
protein metabolic
process
GO:0044260
cellular macromolecule
metabolic process
GO:0044249
cellular biosynthetic
process
GO:0009059
macromolecule biosynthetic
process
GO:0044036 (0.0095)
cell wall
macromolecule metabolic process
93/4004 | 215/16148
GO:0022402 (0.00562)
cell cycle process
140/4004 | 359/16148
GO:0051276
chromosome organization
GO:0048731
system development
GO:0032259
methylation
GO:0043412
macromolecule modification
GO:0006259
DNA metabolic
process
GO:0044267
cellular protein
metabolic process
GO:0034645
cellular macromolecule
biosynthetic process
GO:0006325
chromatin organization
GO:0022403
cell cycle phase
GO:0048513 (0.0301)
organ development
325/4004 | 1016/16148
GO:0043414
macromolecule methylation
GO:0006464
protein modification
process
GO:0006260 (0.0348)
DNA replication
73/4004 | 163/16148
GO:0016568
chromatin modification
GO:0000279
M phase
GO:0043687
post-translational protein
modification
GO:0016569
covalent chromatin
modification
GO:0051322 (0.016)
anaphase
29/4004 | 39/16148
GO:0008213 (0.0182)
protein amino
acid alkylation
47/4004 | 85/16148
GO:0016570
histone modification
GO:0006479 (0.0182)
protein amino
acid methylation
47/4004 | 85/16148
GO:0016571 (0.0157)
histone methylation
45/4004 | 79/16148
GO:0034968 (0.00347)
histone lysine
methylation
45/4004 | 73/16148
GO:0051567 (0.0435)
histone H3-K9
methylation
35/4004 | 57/16148

Figure S7

Supplementary Table S3: Details of primer sequences of selected contigs used for quantitative reverse transcriptase-polymerase chain reaction (qRT-PCR) analysis.

S. No	Gene name	Expression primer sequences	Tm °C	Transcript ID
1.	Regulatory protein NPR3	Fwd 5'-GGACAAGGCCGAGCTTCA-3'<br>Rev 5'-CGCACCGTTGCTCAGAGAA-3'	55	scaffold1047
2.	Flagellin-sensitive 2	Fwd 5'-CGGATGATTTTCCTGTTATAAACTTG-3'<br>Rev 5'-TTCTAGCCTCTTTGACCATGGTAA-3'	55	scaffold23640
3.	ORF2-WRKY	Fwd 5'-CGAGGCATGACACTAGGTTCAG-3'<br>Rev 5'-TTGTTAGATCAAGAGTGATGGTTGGT-3'	55	scaffold9307
4.	Disease resistance protein RPM1	Fwd 5'-CCCAACTCAGCTCATCAGTGAA-3'<br>Rev 5'-TCTAAGATAGCCTCGGATGTTGATC-3'	55	scaffold13306
5.	Disease resistance protein RPS2	Fwd 5'-TTCGCTGTGTCAAACTTCATGA-3'<br>Rev 5'-GTATTGAGTGGCTGGAATGTGAAT-3'	55	scaffold10075
6.	Disease resistance protein RPM1	Fwd 5'-ACACAACAATGGCTAGCGGTAA-3'<br>Rev 5'-ACACCAGGATGGAGATGTTAGTGA-3'	55	scaffold8966
7.	Disease resistance protein RPM1	Fwd 5'-GCACAATTTTCTGATGGATTGATTT-3'<br>Rev 5'-GCTAAGGAATTGAATCATGAAGAGTCT-3'	55	C218622
8.	Disease resistance protein RPS2	Fwd 5'-TGGTGAGCATTGCAGAATCATT-3'<br>Rev 5'-GGTTCACCAAGTCTGATATGGAATT-3'	55	scaffold24649
9.	Xyloglucan:xyloglucosyl transferase	Fwd 5'-GGCTTTGCTCCAATCAATCTTT-3'<br>Rev 5'-ACACTTTGGGAAGCCGATGA-3'	55	scaffold23674
10.	Disease resistance protein RPS2	Fwd 5'-CAACATAAAGATAGGCATTCATGGAA-3'<br>Rev 5'-TAAGAAGTCGTAACTCAACTCCAGAGA-3'	55	CL39Contig5
11.	Disease resistance protein RPM1	Fwd 5'-TCGCGGCTTACATCATATTCAA-3'<br>Rev 5'-GCGGCTAATCGTAGTAAATTTCACT-3'	55	scaffold17813
12.	Disease resistance protein RPS2	Fwd 5'-TGGAACTCATTAAATTGGCTAGCAT-3'<br>Rev 5'-TCTCACGCATCTCTTGCTAAGTG-3'	55	scaffold6345
13.	Brassinosteroid insensitive1	Fwd 5'-GAATCGCGATGTTTTGTGTCA<br>Rev 5'-GAATCGCGATGTTTTGTGTCA	55	scaffold24185

Tm°C: melting temperature

qRT-PCR conditions :

The PCR amplification program was as follows: 95°C for 10 min; 35 cycles of 95°C for 30s, Tm°C for 30 sec and 72°C for 30s; followed by a melting-curve program of 95°C for 1min, 55°C for 30s and 95°C for 30s.

Supplementary Tables Legends

Supplementary Table S1: Annotations details of NR data derived from RNA-seq data of RG and SG during BB transition in Tea: Table S1a: GO annotation Molecular function and Blastx; Table S1b: GO annotation Cellular Component and Blastx; Table S1c: GO annotation Biological Processes and Blastx; Table S1d: KEGG annotation and Blastx; Table S1e: Enzyme Commission and Blastx; Supplemenatary_TableS1f: Transcription factors classification and Blastx.

Supplementary Table S2: EdgeR based differentail gene expression of 149 defense related transcipts in RNA seq data of RG and SG during BB transition in Tea

Supplementary Table S3: qRT-PCRanalysis; Details of primer sequences of selected transcripts used for quantitative reverse transcriptase-polymerase chain reaction (qRT-PCR) analysis.

Supplementary Figure legends

Supplementary Figure S1: Biological processes GO enrichment analysis of major pathways during BB progression at S1_RG.

Supplementary Figure S2: Biological processes GO enrichment analysis of major pathways during BB progression at S1_SG.

Supplementary Figure S3: Biological processes GO enrichment analysis of major pathways during BB progression at S2_RG.

Supplementary Figure S4: Biological processes GO enrichment analysis of major pathways during BB progression at S3_RG.

Supplementary Figure S5: Biological processes GO enrichment analysis of major pathways during BB progression at S3_SG.

Supplementary Figure S6: Biological processes GO enrichment analysis of major pathways during BB progression at S4_RG.

Supplementary Figure S7: Biological processes GO enrichment analysis of major pathways during BB progression at S4_SG.
